# Editorial: Molecules of the extracellular matrix as cancer targets

**DOI:** 10.3389/fonc.2022.1034646

**Published:** 2022-09-20

**Authors:** Nikos K. Karamanos, Martin Götte, Alberto Passi

**Affiliations:** ^1^ Biochemistry, Biochemical Analysis & Matrix Pathobiology Res. Group, Laboratory of Biochemistry, Department of Chemistry, University of Patras, Patras, Greece; ^2^ Department of Gynecology and Obstetrics, Münster University Hospital, Münster, Germany; ^3^ Department of Medicine and Surgery, University of Insubria, Varese, Italy

**Keywords:** extracellular matrix, cancer, proteoglycans, glycosaminoglycans, metalloproteinases, heparanase, tenascins, molecular targets

Extracellular Matrices (ECMs) are potent 3D-milieux contributing to the structural integrity of tissues and organs (1). The complex interactions between cells and the surrounding matrix but also within ECM are pivotal for normal development and tissue homeostasis (2). ECM networks, formed by cooperating macromolecules and active effectors, also modulate cell–matrix interactions, cell morphology, growth and function. The ECMs building blocks comprise proteoglycans (PGs) and glycosaminoglycans (GAGs), collagens, fibronectin, tenascins, elastin as well as cell-surface receptors such as integrins, CD44, discoidin domain receptors (DDRs), and receptor tyrosine kinases (RTKs) (3). ECMs function as intermediates between the cells in organs and tissues by regulating multiple inside-out or outside-in signals.

ECM macromolecules play key regulatory roles in gene expression, cell signaling and functions in tissue properties affecting human pathophysiology. Cell-matrix interactions and matrix remodeling are crucial for development, tissue regeneration, as well as disease development and progression. Interactions within the ECMs are quite convoluted and responsible for producing commands to determine vital cellular properties, such as proliferation, motility, autophagy, adhesion, and differentiation (4–7). Building biomolecular networks allows for the conjecture of individual cell properties through association of matrix components, and assists the discovery of targeted therapeutics and their use in drug design (8). It is worth noticing that ECM assembly and organization is constantly adapted based on distinct biomechanical signals.

ECM as a regulatory partner in tissue homeostasis, cancer development and progression, is an emerging field of research, and expression of matrix macromolecules and ECM structural integrity are significantly altered in cancer. Research in the field is focused not only to the extracellularly secreted macromolecules, but also key intracellular, pericellular and basement membrane molecules. Considering its relevance for disease development, the ECM-related field, although emerging, has been underestimated in terms of studies on the mechanistic links behind matrix and several diseases, its prognostic value, and the designing of novel comprehensive treatment strategies.

Recent advances in ECM research have shown that several ECM effectors could be useful potential markers for diagnostic purposes and pharmacological targeting of cancer. Accumulative knowledge has also demonstrated that apart from their action at a cellular level, ECM effectors within their interaction networks could be useful modern biotechnological tools for drug delivery and targeted therapies. Recent research in this area will improve our understanding on the underlying mechanisms involved in cancer development and progression. Advancement in knowledge around ECM has the possibility to guide the development of novel diagnostic tools, innovative therapies, and biomarkers, improving disease management.

This Research Topic collection consists of 26 papers, 18 original research and 8 review articles, co-authored by 166 researchers in the field, and aims to provide the latest advances and insights in the field of ECM in cancer with focus on the ECM roles in cell signaling and cellular functions (proliferation, differentiation, migration, morphology, epithelial-to-mesenchymal transition, angiogenesis, autophagy, etc), pharmacological targeting/treatment and bioengineering approaches.

PGs significantly contribute to the cell signaling and immune responses. The extracellular small leucine-rich proteoglycans, decorin and biglycan, affect tumor growth and progression. Diehl et al. present and critically discuss the complex roles of decorin and biglycan signaling in tumor biology and their potential novel therapeutic implications. Soluble decorin and biglycan modulate key processes vital for tumor initiation and progression, such as autophagy, inflammation, cell-cycle, apoptosis, and angiogenesis. The adaptive nature of a malignant neoplasm is also a major challenge for the development of effective anti-oncogenic therapies.

The RTKs-mediated cell signaling, and integrin matrix receptors have well-established roles in tumor growth and propagation. Rapraeger discussed the syndecans as key molecules in this context. The syndecans, a family of cell membrane heparan sulfate proteoglycans (HSPGs), acting as matrix receptors, organize RTKs and integrins into functional units. Synstatins, peptide mimetics of the docking motifs in the syndecans, prevent assembly of these receptor complexes, block signaling activities and are highly effective against tumor cell invasion, survival and angiogenesis. Syndecan-1, on the other hand, has also been correlated with the development of cervical carcinoma. Syndecan-1 serves as a matrix receptor and coreceptor for receptor tyrosine kinases and additional signaling pathways. The loss of syndecan-1 expression is associated with low differentiation of cervical carcinoma and with an increased rate of lymph node metastases. Hilgers et al. analysed the clinical impact of syndecan-1 expression by analysis of public gene expression datasets and studied the effect of an overexpression of syndecan-1 in the human cervical carcinoma HeLa cells. High syndecan-1 expression correlated with a poor prognosis, suggesting its important role in cervical cancer progression. The reduced syndecans-1-dependent cell motility was linked to the Rho-GTPase signaling pathway. Conclusively, in cervical cancer syndecan-1 modulates pathogenetically relevant processes, which depend on the membrane-association of syndecan-1.

Serglycin, a well-known intracellular PG, is highly expressed by immune cells., It has been recently demonstrated that serglycin is also expressed by several other cell types, such as endothelial cells, muscle cells, and multiple types of cancer cells. Tellez-Gabriel et al. demonstrated that serglycin expression is upregulated in TGF-β induced epithelial-mesenchymal transition (EMT) and provided evidence that is a significant EMT marker gene. Notably, serglycin is more expressed by breast cancer cell lines with a mesenchymal phenotype as well as the basal-like subtype of breast cancers. Authors also found that serglycin is highly expressed by infiltrating immune cells in breast tumor tissue.

The extracellular matrix proteoglycan SPOCK1 contributes to the development and progression of cancers. SPOCK1 present in non-tumorous hepatocytes at low concentrations, promotes the development and progression of malignant hepatocellular tumors. It is worth noticing that syndecan-1, the major proteoglycan of the liver, and SPOCK1 are in inverse correlation. Váncza et al. showed that SPOCK1 downregulation of hepatoma cell lines upregulated p21 and p27 and interfered with pAkt and CDK4 expression. SPOCK1 in the liver cancer cells altered MAPK signaling and downregulated several members of the Src family, all related with the aggressive potential of the hepatoma cells. Therefore, SPOCK1 enhancement in the liver is an active contributor to hepatocarcinogenesis and cancer progression.

The fine structural features of heparan sulfate (HS) chains, including length and sulfation patterns, are crucial for the biological roles displayed by HSPGs, as these features determine binding affinities and selectivity. Marques et al. address in their review the regulatory mechanisms underlying HS biosynthesis and provide insights on the impact of different HS structural epitopes as well as on the effects of deregulated expression of HS modifying enzymes in the development and progression of cancer. The clinical potential of HS biosynthetic enzymes as novel targets for cancer therapy are also presented and discussed. HS chains of endothelial cell PGs interact with the major angiogenic factors, regulating blood vessels´ formation. Melo et al. investigated the effect of a selected HS-binding peptide in angiogenesis and tumor progression. The HS-binding peptide showed a higher affinity for N-sulfated heparin. The HS-binding peptide inhibits the proliferation of human endothelial umbilical cord cells by modulation of FGF-2 and significantly decreases the tube formation of endothelial cells and capillary formation of aorta, the formation of sub-intestinal blood vessels and the tumor size in zebrafish embryos.

Another type of sulfated GAGs, chondroitin sulfate (CS) differs from HS in structural unit hexosamine component and the fine sulfation features. CS chains may also regulate cancer cell properties. CS enhances the invasive activity of the human triple-negative breast cancer (TNBC) cell line MDA-MB-231, but its molecular mechanism remains unclear. Nadanaka et al. demonstrated that CSs bind to ROR1 in the presence of WNT5A. The invasive activity of MDA-MB-231 cells enhanced by CSs was completely suppressed by ROR1 knockdown and knockdown of the CS biosynthesis inhibited invasive activity, even in the presence of ROR1, suggesting that CS is required to induce an ROR1-dependent, aggressive phenotype. Taking into consideration that CS promotes cancer aggressiveness through the ROR1−JNK axis, this study opens a new area of research to help pharmaceutical targeting in TNBC. Tsidulko et al. investigated off-target negative effects of the systemic chemotherapy on glycosylated components of the brain ECM. Using an elaborated glioblastoma multiforme (GBM) relapse animal model, authors demonstrated that healthy brain tissue resists GBM cell proliferation and invasion, thereby restricting tumor development. Adjuvant chemotherapy with temozolomide induced changes in composition and content of brain PGs resulted in the accelerated adhesion, proliferation, and invasion of GBM cells into brain organotypic slices *ex vivo* and more active growth and invasion of experimental xenograft GBM tumors in SCID mouse brain *in vivo*. Notably, degradation of CS chains was identified as a key event responsible for the observed functional effects. As a future perspective, authors suggest that ECM-targeted supportive therapy might mitigate the negative off-target effects of the adjuvant GBM treatment and increase the relapse-free survival of GBM patients.

The well-known high molecular weight non-sulfated GAG, hyaluronan (HA), plays pivotal roles in tissue homeostasis, regeneration, several cell functional properties, but also has been related to various pathological conditions including inflammation and cancer. We are delighted that Takabe et al. reviewed the role of HA in the progression of cutaneous melanoma, an aggressive type of skin cancer. The expansion of melanoma is affected by the ECM surrounding the tumor together with immune cells. In early disease stages, HA is the major matrix component in cutaneous melanoma microenvironment. In advanced melanoma, HA content decreases due to altered synthesis and degradation, correlating with poor prognosis. This review focuses on HA matrix in cutaneous melanoma and how the changes in HA metabolism affect the progression of melanoma. HA, as one of the most abundant molecules in the TME, is also often found to coat extracellular vesicles (EVs). EVs derived from plasma membrane tentacles of cancer cells are crucial for migration, such as filopodia, and are abundant in tumor niches. Thus, it is possible that HA and HA-coated EVs have a cooperative role in promoting migration. Aaltonen et al. compared the HA synthesis, EV secretion and migratory behavior of normal and aggressive MCF10 series breast cell lines. Authors demonstrated that EVs left behind by tumor cells during migration are strongly positive for CD9. A leader-follower behavior was significantly decreased upon removal of pericellular HA, indicating that HA promotes the pathfinding behavior of follower cells. The results suggest the orchestrated role of HA, EVs and other extracellular cues in coordinated migration and pathfinding behavior of follower cells.

It is widely accepted that the TME, particularly the ECM, plays an essential role in tumor development through the interaction with specific protein-membrane receptors. One of the most relevant proteins in this context is the transmembrane protein CD44. The role of CD44 in tumor progression, invasion, and metastasis has been well established in many cancers. Fernández-Tabanera et al. summarize its role in sarcomas. CD44 is overexpressed in most sarcomas and exhibits a direct effect on tumor progression, dissemination, and drug resistance. CD44 is a useful prognostic and diagnostic marker (CD44v6 isoform) in osteosarcoma. HA-functionalized liposomes therapy has become an excellent CD44-mediated intracellular delivery system for osteosarcoma. Further research involving the specific role of CD44 in different sarcoma subgroups could provide a more innovative perspective for novel therapies and future clinical trials. Cancer-initiating cells (CICs) drive colorectal tumor growth and interact with multiple cell types within TME, including cancer-associated fibroblasts (CAFs). Chemoresistance in colorectal CICs involves the sustained activation of multiple drug resistance and WNT/β-catenin signaling pathways, as well as of alternatively spliced-isoforms of CD44 containing variable exon-6 (CD44v6). However, the mechanisms underlying sustained WNT/β-catenin signaling have remained elusive. Ghatak et al. investigated the interplay between the CICs and the chemotherapeutic FOLFOX that creates the persistent tumorigenic properties of colorectal CICs and stimulates the microenvironmental factors derived from the CAFs. Using biochemical, molecular and cell biology approaches, cell signaling pathways evaluation and *in vivo* mouse authors demonstrated that the interplay between chemotherapy-activated CAFs and CICs expressing cd44v6 promotes colon cancer resistance. These findings have crucial clinical implications suggesting that more specific therapeutic approaches required to block a chemotherapy induced remodeling of a TME that acts as a paracrine regulator to enrich CD44v6 (+) in colorectal CICs. In the subsequent study Misra et al. provide evidence that targeting CD44v6-mediated LRP6/β-catenin-signaling and drug efflux may represent a novel approach to overcome FOLFOX resistance and inhibit tumor progression in colorectal CICs. Sustained drug resistance in colorectal CICs is mediated by overexpression of CD44v6, which is both a functional biomarker and a therapeutic target in colorectal cancer. Cold atmospheric plasma (CAP) has been proposed as an emerging anti-cancer treatment modality. Aggelopoulos et al. investigated the effects of direct and indirect CAP treatment on breast cancer cells of different estrogen receptor (ER) status. CAP treatment induced intense phenotypic changes and apoptosis in both ER+ and ER- cells. Interestingly, CAP significantly reduced CD44 protein expression, while differentially affected the expression of proteases and inflammatory mediators. Collectively, the findings of the present study suggest that CAP suppresses breast cancer cell growth and regulates several effectors of the TME and thus it could represent an efficient therapeutic approach for distinct breast cancer subtypes.

DDR1, a collagen receptor and tyrosine kinase, has emerged as an important player in cancer. Sirvent et al. review new DDR1 functions in tumor dormancy following dissemination, immune exclusion and therapeutic resistance induced by stromal collagens deposition. The signaling mechanisms behind these tumor activities and the therapeutic strategies aiming at targeting these collagens-dependent tumor responses as well as future perspectives are also presented and discussed.

Integrin β superfamily members (ITGBs) play important roles in various biological processes and are associated with carcinogenic effects in several malignancies. However, the expression and prognostic values of ITGBs and potential mechanism in gastric cancer (GC), a highly complex and heterogeneous disease, remain largely unclear. Liu et al. evaluated the ITGBs expression profiles in GC using various bioinformatic databases. Authors found that ITGB1-2 and ITGB4-8 are significantly higher in GC, high ITGB5 expression contributes to a poor prognosis and its expression is significantly associated with the ECM organization, cell-substrate adhesion, and ossification. Conclusively, ITGB5 may function as a valid biomarker of prognosis, and high expression of ITGB5 predicts poor prognosis for in GC and might be a potential target of precision therapy. Integrin α11β1 is a collagen-binding integrin. The expression of the α11 is upregulated in CAFs in various human neoplasms. Martínez-Nieto et al. investigated α11 expression in human cutaneous squamous cell carcinoma (cSCC) and in benign and premalignant human skin lesions and monitored its effects on cSCC development. Integrin α11 expression was significantly upregulated in the desmoplastic tumor stroma of human and mouse cSCCs, and the highest α11 expression was detected in high-grade tumors. Authors suggest that α11β1 operates in a complex interactive TME to regulate ECM synthesis and collagen organization and thus foster cSCC growth. Advanced experimental models will help to define the exact roles and molecular mechanisms of stromal α11β1 in skin tumorigenesis.

Targeting tumor-specific ECM molecules and stromal cells or disrupting aberrant mesenchyme-cancer communications might normalize the TME and improve cancer treatment outcome. The tenascins are a family of large, multifunctional extracellular glycoproteins consisting of four members. Tenascin-C and -W are currently the most promising candidates for exploitability and clinical use as they are highly expressed in various tumor stroma with relatively low abundance in healthy tissues. Tucker and Degen review the expression of all four tenascins in tumors, followed by a more thorough discussion on tenascin-C and tenascin-W focusing on their oncogenic functions and their potential as diagnostic and/or targetable molecules for anti-cancer treatment purposes.

Cells recognize and communicate with the surrounding cells and ECM to maintain homeostasis. ECM remodeling is critical for the maintenance of normal functionality. The cellular microenvironment is optimized for the proper functioning of the tissues and organs. Nevertheless, under pathological conditions like cancer, ECM remodeling ceases to be subject to control resulting in disease initiation and progression (9, 10). The actions of proteolytic enzymes such as matrix metalloproteinases (MMPs) as well as non-proteolytic ones such as heparanase (HPSE) alter the ECM composition, can modify the stability and functions of the ECM and drive disease initiation and progression. When cancer arises, the cellular microenvironment is modified to optimize its malignant growth, evading the host immune system and finding ways to invade and metastasize to other organs. Indeed, cancer progression relies on not only the performance of cancer cells but also the surrounding microenvironment. Itoh in his mini review discusses the current understanding of proteolytic modification of the TME signals during cancer progression.

Among glycosidic enzymes, HPSE, the mammalian endoglycosidase degrading HS, has been extensively studied the last decade. It regulates EMT and cancer stem cell properties and involved in prostate cancer progression. Cancer stem cells (CSCs) or cancer-initiating cells contribute to the initiation and tumor growth, the metastatic potential and drug resistance. Masola et al. evaluated two prostate cancer cell lines (DU145 and PC3) following HPSE silencing and overexpression. Expression of EMT and stemness markers was evaluated. The novel findings support a new mechanism of HPSE action in sustaining prostate cancer growth and diffusion and highlight the importance of HPSE as a potential pharmacological target.

Interactions among cancer cells and TME are orchestrated by the ECM contributing to fundamental processes of breast cancer progression. Estrogen receptors (ERs) have pivotal roles in the development and progression of TNBC. Early studies have correlated ERβ expression in tumor sites with a more aggressive clinical outcome (11). Piperigkou et al introduced the functional role of ERβ suppression following isolation of monoclonal cell populations of MDA-MB-231 breast cancer and demonstrated that clone selection results in suppression of the aggressive cell functional properties by transforming their morphological characteristics, eliminating the mesenchymal-like traits. shERβ MDA-MB-231 cells undergo universal matrix reorganization, pass on a MET transition state and prevent tumorigenesis *in vivo.* These novel findings highlight the promising role of ERβ targeting in future pharmaceutical approaches for managing the high metastatic potential of TNBC.

A major and urgent issue is to identify diagnostic biomarkers of ovarian cancer (OC) to understand the underlying mechanism. Yang et al. studied cell functional properties and EMT using bioinformatic tools, biochemical and cell molecular biology approaches. LncRNA RP11-499E18.1 coexists in the nucleus and cytoplasm of OC cells. Its overexpression suppressed OC cell proliferation, migration, and colony formation, as well as SOX2 nuclear translocation. Interestingly, RP11-499E18.1 downregulated, while PAK2 and SOX2 was upregulated in OC tissues and cells. The new concept of this study indicates that RP11-499E18.1 might be a valuable diagnostic biomarker in OC and might play its tumor suppressor roles *via* regulation of the RP11-499E18.1–PAK2–SOX2 axis.

The low-density lipoprotein receptor-related protein 1 (LRP1), an endocytic receptor, mediates the clearance of ECM and regulates the expression of matrix receptors. However, the underlying mechanisms remains partial in the frame of cancer cells interaction with matricellular substrates. Langlois et al. identified that LRP1 downregulates calpain activity and calpain 2 transcriptional expression in an invasive thyroid carcinoma cell model, limiting cell-matrix attachment strength. Authors found that LRP-1 exerts a dual mode of control of calpain activity fine-tunes carcinoma cell spreading and suggest an additional and innovative intracellular mechanism which demonstrates LRP-1 pro-motile action in thyroid cancer cells.

Vinculin as a focal adhesion protein play critical role in cell attachment and detachment during migration. Metsiou et al. evaluated the spreading rate and the adhesion strength between breast cancer cells and ECM prior to and post-treatment with anti-tumor agents using tamoxifen treatment for ER+ breast cancer cells, and trastuzumab and pertuzumab for HER2+ cells. Post-treatment spreading rate was significantly decreased in both types of breast cancer, suggesting a lower metastatic potential. Treated cells required greater adhesion forces to detach from the ECM. Post-detachment and post-treatment vinculin levels were increased, indicating tighter cell–ECM junctions, limiting the probability of cell motility and migration.

Although 2D *in vitro* cell culture studies are common in the cancer research, reliable biomimetic 3D models are needed to ensure physiological relevance. Iazzolino et al. hypothesized that decellularized xenograft tumors can serve as an optimal 3D substrate to generate a top-down approach for *in vitro* tumor modeling. Authors found that samples decellularized from TNBC basal-like subtype xenograft models had different ECM compositions compared to the rest of the xenograft tumors tested. The *in vitro* recellularization of decellularized ECM (dECM) yields tumor-type–specific cell behavior in the TNBC context. Authors suggest that dECM is a feasible substrate for reseeding purposes, thereby promoting tumor-type–specific cell behavior, a proof-of-concept for further potential generation of patient-specific *in vitro* research models.

## Author contributions

All authors listed have made a substantial, direct, and intellectual contribution to the work and approved it for publication.

## Acknowledgments

Guest editors appreciate the kind participation of scientists contributed in this Research Topic with their research and review articles. We also thank the valuable help of Dr. K. Kyriakopoulou for the initial drafting of the editorial.

## Conflict of interest

The authors declare that the research was conducted in the absence of any commercial or financial relationships that could be construed as a potential conflict of interest.

## Publisher’s note

All claims expressed in this article are solely those of the authors and do not necessarily represent those of their affiliated organizations, or those of the publisher, the editors and the reviewers. Any product that may be evaluated in this article, or claim that may be made by its manufacturer, is not guaranteed or endorsed by the publisher.
